# Nanoelectronics-enabled reservoir computing hardware for real-time robotic controls

**DOI:** 10.1126/sciadv.adu2663

**Published:** 2025-03-26

**Authors:** Mingze Chen, Xiaoqiu An, Seung Jun Ki, Xirong Liu, Nihal Sekhon, Artyom Boyarov, Anushka Acharya, Justin Tawil, Maxwell Bederman, Xiaogan Liang

**Affiliations:** Department of Mechanical Engineering, University of Michigan, Ann Arbor, MI 48109, USA.

## Abstract

Traditional robotic vehicle control algorithms, implemented on digital devices with firmware, result in high power consumption and system complexity. Advanced control systems based on different device physics are essential for the advancement of sophisticated robotic vehicles and miniature mobile robots. Here, we present a nanoelectronics-enabled analog control system mimicking conventional controllers’ dynamic responses for real-time robotic controls, substantially reducing training cost, power consumption, and footprint. This system uses a reservoir computing network with interconnected memristive channels made from layered semiconductors. The network’s nonlinear switching and short-term memory characteristics effectively map input sensory signals to high-dimensional data spaces, enabling the generation of motor control signals with a simply trained readout layer. This approach minimizes software and analog-to-digital conversions, enhancing energy and resource efficiency. We demonstrate this system with two control tasks: rover target tracking and drone lever balancing, achieving similar performance to traditional controllers with ~10-microwatt power consumption. This work paves the way for ultralow-power edge computing in miniature robotic systems.

## Teaser

Layered semiconductors enable a real-time, energy-efficient reservoir computing system for robotic control.

## INTRODUCTION

Artificial intelligence has already exceeded human capabilities in various tasks, including pattern/voice recognition and language processing (*1*). However, to implement the robotic functionalities involving complicated dynamic processes, the conventional methodologies always involve the tasks for handling large amounts of the temporal information carried by time-series sensory signals. These methodologies are approaching the physical limits of conventional computing and controlling systems based on digital electronics and software/firmware implementations, which is ultimately imposed by the failure of Moore’s rule (*2*, *3*). One of the critical indicators for this issue is that the advancement of these conventional control systems results in formidable power consumption and system complexity/size for handling the time-series data from increasingly complex sensors. This issue seriously retards the development of the control systems for increasingly sophisticated robotic vehicles and miniature mobile robots. Toward tackling this grand challenge, various hardware-based artificial neural networks (ANNs) have been developed, seeking to overcome the intrinsic limits of software-based counterparts and create energy-efficient computing systems through emulating the biological neural networks in human brains where both computation and memory processes are performed through synapses (connections) between neurons (*4*, *5*). For example, memristor-based ANNs have shown orders of higher energy efficiency than that of the traditional digital computing methods in multiple tasks (*6*–*9*). This is because the input data can be processed in a parallel and purely analog fashion via fundamental physics laws (i.e., Ohm’s law) using the analog resistive switching behaviors of memristors. More specifically, in these works, the memristor—a two-terminal electronic device—can be modulated to different conductance states. Depending on their retention characteristics of conductance states, memristors can be categorized into two groups: nonvolatile memristors (NVMs) and dynamic memristors (DMs). NVMs, with long-term memory of resistance change, have been widely used as the synaptic nodes of the ANNs dedicated to the tasks involving complex matrix calculation, such as pattern recognition, classification, regression, and function approximation (*4*, *10*–*12*). However, these ANNs are not suitable for processing the data involving temporal information because they mainly function as feedforward networks, lacking device physics schemes for mapping or filtering the data with different temporal contexts (*13*–*15*).

The recurrent neural network (RNN) framework, in which the neurons with recurrent connections can learn from previous time steps using feedback loops, has been designed for performing the tasks involving time-sequential data processing (*16*–*18*). However, the software-based RNN implementation typically demands extensive energy consumption for exploding or vanishing gradients in recurrent structures during training stages (*19*, *20*), while hardware-based RNN systems can be hardly constructed using regular memory devices or aforementioned NVMs because the steady conductance states of these devices can hardly extract the temporal information components in an analog way without software involvement. To address these issues, different dynamic physical device systems, including DMs (*6*, *21*–*23*), have been developed and investigated, seeking to realize the hardware implementation of RNN schemes. One of the important RNN schemes under this investigation is reservoir computing (RC). RC is a machine learning framework that aims to efficiently process temporal data using a fixed, randomly initialized RNN termed the reservoir, and, therefore, it features a low training cost. In an RC system, its reservoir with short-term memory characteristics is used to nonlinearly project the temporal inputs into a high-dimensional feature space, which is represented by the dynamic state of the neurons (or dynamic nodes) in the reservoir. Mathematically, this nonlinear mapping process is performed through the neuron activation function, typically a sigmoid function, which transfers the complicated input signals into linearly separable signals in the form of reservoir states (*4*, *24*–*26*). Therefore, unlike traditional RNNs, an RC network only needs the training for a readout layer to correlate the state vectors of the reservoir to the expected output signals, making it computationally efficient and easy to implement (*24*, *27*). In addition, the low training cost and computation simplicity make RC systems an ideal substitution for other heavy-computation algorithms and control systems (*28*–*30*).

When serving as the neuron units in an RC network, DMs can perform continuous and nonlinear transformations of the input signals, such as thresholding and exponential correlation (*6*, *23*). Furthermore, the short-term memory characteristics of DMs can enable neuron-like temporal dynamics with higher-order complexity compared to NVM-based systems (*31*, *32*). This unique signal transformation capability and short-term memory properties make DMs very suitable for serving as physical neurons in RC networks. DM arrays have been used to construct the RC systems for the tasks including speech recognition (*21*, *33*), signal forecasting (*21*, *31*, *33*), and wave classification (*6*). However, in these works, the continuous input signals need to be converted to discrete time-series pulses or spikes through sampling or mask processes (still similar to analog-to-digital conversion processes), and these RC systems typically generate discrete outputs to accomplish classification-style missions, which have not fully leveraged the dynamic aspects of these DM-based RC systems. A potentially better niche application of these hardware-based RC systems is to control highly dynamic systems, such as robotic vehicles. To enable this application, real-time control signals need to be constantly produced through dynamic extraction of a broad range of temporal context information components (i.e., current, historical, and trend readings) from the input analog sensory signals. Furthermore, in an RC system dedicated to this mission, the sensory signal components with different temporal aspects or contexts need to be nonlinearly transformed and differentiated through different activation functions. To set up a software-based RC system, these activation functions can be easily programmed to fulfill the mathematical requirements of an RC framework, while the hardware-based RC implementation demands specific state-switching characteristics of the physical devices involved in the system. Nevertheless, it is very challenging to use the aforementioned DM arrays to realize dynamic extraction of highly diversified temporal information components and nonlinear signal mapping because they are manufactured with similar switching properties. Therefore, a holistic memristive reservoir network, which involves a set of artificial neurons with intrinsically diverse short-term memory characteristics, is highly desirable for realizing hardware-based neuromorphic control of dynamic systems.

Here, we report a memristive network–based reservoir made from layered semiconducting Bi_2_Se_3_ capable of processing analog temporal signals and implementation of this hardware-based RC device for controlling two robotic systems with reduced power consumption. Specifically, we fabricate a Bi_2_Se_3_-based memristive network that serves as the hardware-based RC unit using multiplexing rubbing-induced site-selective (RISS) deposition. This RISS method, as demonstrated in our previously published works, can directly produce arbitrarily designed two-dimensional (2D) material patterns without additional lithographic and etching processes (*34*–*36*). The presented RISS-produced Bi_2_Se_3_ memristive network can function as a reservoir rich in dynamic states, which are represented by the internal resistance states of all memristive channels in the reservoir network. These dynamic reservoir states can be quantified through multiple neuron pathways, each of which exhibits unique nonlinear memristive switching and short-term memory characteristics in response to time-sequential voltage signals. These diversified dynamic effects allow the RC system to extract temporal information components from the input sensory signals by mapping the sensor readings with these components to high-dimensional reservoir states, which have yet to be demonstrated by our understanding but are critical for dynamic system control applications. After the RC-enabled mapping process, a simple software-based readout layer with pretrained weight parameters is used to correlate the internal reservoir state vectors with the anticipated output control signals. In this work, we implement our hardware-based RC system to physically emulate the controlling behavior of a proportional-integral-derivative (PID) controller in two robotic vehicle control tasks: a robotic rover to perform target-tracking navigation and a drone motor to keep a dynamic lever in balance. For both tasks, the RC-controlled systems exhibit very similar dynamic characteristics as the counterparts controlled by the PID scheme, which is quantitatively indicated by relatively low normalized root mean square error (NRMSE) values of 0.11 and 1.25 for rover navigation and lever balancing tasks, respectively. In addition, in comparison with previously reported hardware-based RC systems, our memristive RC system based on layered semiconducting materials exhibits a much lower operation power of 12.5 μW and also holds a high potential for enabling miniaturization of the robotic vehicle controller systems (*6*, *21*, *33*).

## RESULTS

### Fabrication of Bi_2_Se_3_-based memristive reservoir networks

**Fig. 1.**
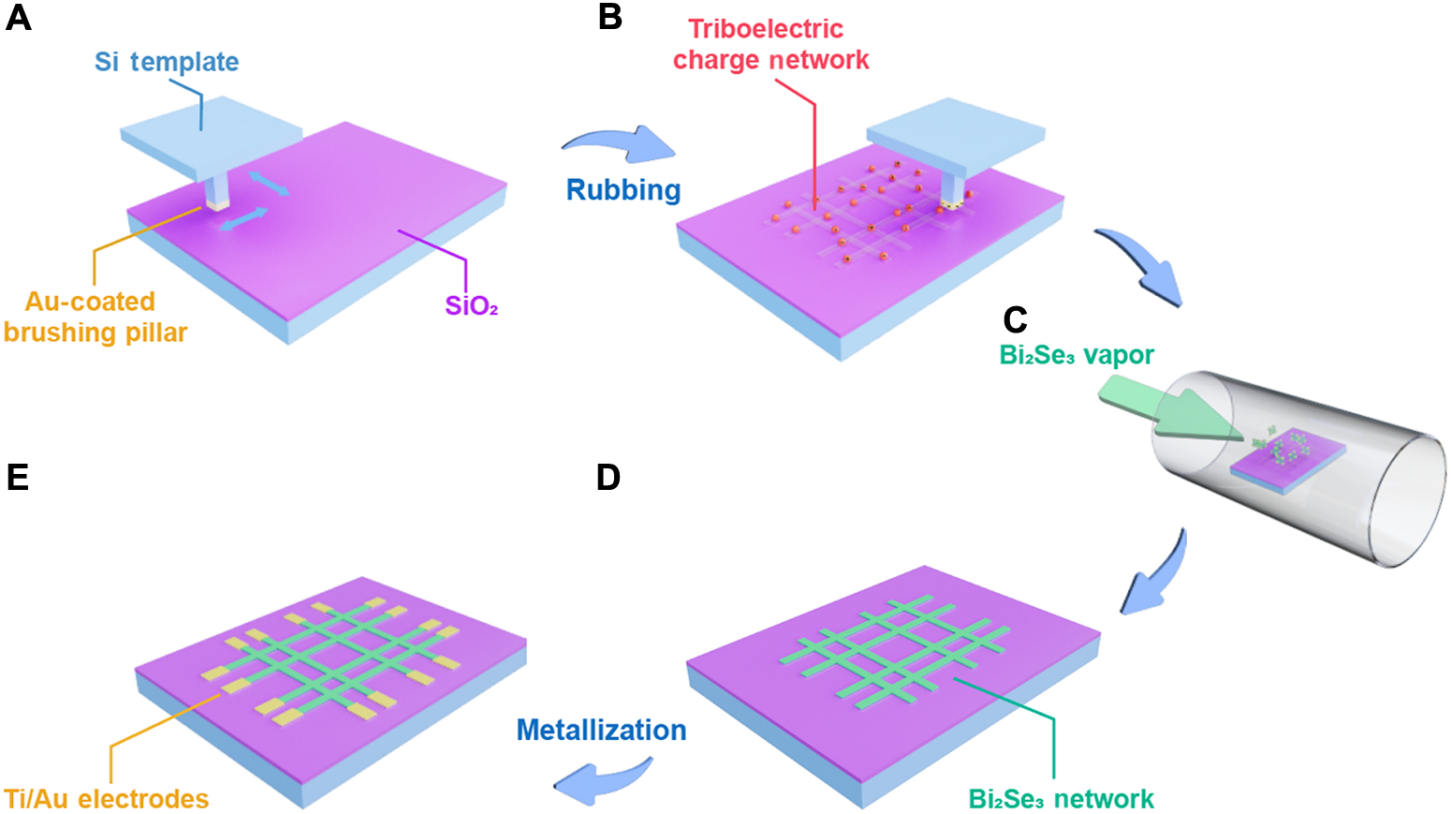
Schematics of the RISS process for making a Bi_2_Se_3_ memristive reservoir. (**A**) Engagement of a Si template bearing an Au-coated brushing pillar to a SiO_2_/Si substrate. (**B**) Formation of a triboelectric charge pattern with a tic-tac-toe frame shape through a stage-controlled rubbing process. (**C**) Loading of the substrate with the triboelectric charge pattern in a furnace for vapor deposition of Bi_2_Se_3_ features. (**D**) Site-selective deposition of Bi_2_Se_3_ channels in the rubbed areas on the SiO_2_/Si substrate. (**E**) Fabrication of the electrodes to define the neuron channels of the Bi_2_Se_3_ memristive reservoir.

Figure 1 (A to E) schematically illustrates the fabrication process of a Bi_2_Se_3_-based memristive reservoir network on a Si/SiO_2_ substrate using the RISS deposition process (*36*). First, the SiO_2_-coated Si substrate is selectively rubbed by a Si template bearing a single Au-coated rubbing pillar (Fig. 1A). This process, performed on a 3D moving stage system, generates a triboelectric charge pattern with tic-tac-toe frame shape in the substrate regions rubbed by the rubbing pillar (Fig. 1B) (*37*, *38*). Afterward, the rubbed substrate, carried with triboelectric charge patterns, is loaded into a tube furnace for depositing Bi_2_Se_3_ patterns through a vapor deposition process (Fig. 1C). Because of the presence of triboelectric charge patterns, the vaporized Bi_2_Se_3_ molecules are polarized by the triboelectric field into dipoles, which are attracted and further nucleated within these charged areas, site-selectively producing Bi_2_Se_3_ network features (Fig. 1D). As reported in our previous works, these RISS-produced Bi_2_Se_3_ channels are rich in Se vacancies and exhibit prominent memristive switching behaviors with short-term plasticity (*34*). Therefore, the network consisting of these interconnected Bi_2_Se_3_ channels is expected to have a huge number of dynamic resistive states. Last, the Ti/Au electrodes are fabricated on top of the as-deposited Bi_2_Se_3_ network by photolithography, followed by metallization to construct a memristive reservoir network (Fig. 1E). The network pattern dimensions, including the spacing, length, and width of interconnected Bi_2_Se_3_ channels, are the arbitrary values selected in a reasonable range (30 to 100 μm), which incorporate some extent of the randomness into the memristive reservoir. Furthermore, the thermal and electrical stability of Bi_2_Se_3_ equips this memristive reservoir with repeatable and robust signal response behaviors (*39*, *40*).

The Bi_2_Se_3_-based memristive channels in our RC network system need to have large dynamic switching ranges for generating rich dynamic states capable of mapping temporal information components in the input signals to high-dimensional data spaces. Our previously published work has shown that the Bi_2_Se_3_ memristors fabricated by mechanical layer exfoliation, followed by lithography and plasma etching processes, exhibit much smaller dynamic ranges (~0.04%) for conductance modulation, while the RISS-produced memristors typically have dynamic modulation ranges of >15%, which is attributed to the higher Se vacancy concentration induced by RISS processes (*34*). Therefore, for the RC applications, the RISS method is highly desirable in comparison with regular lithography and etching steps.

### Material characterizations of RISS-produced Bi_2_Se_3_ network channels

**Fig. 2.**
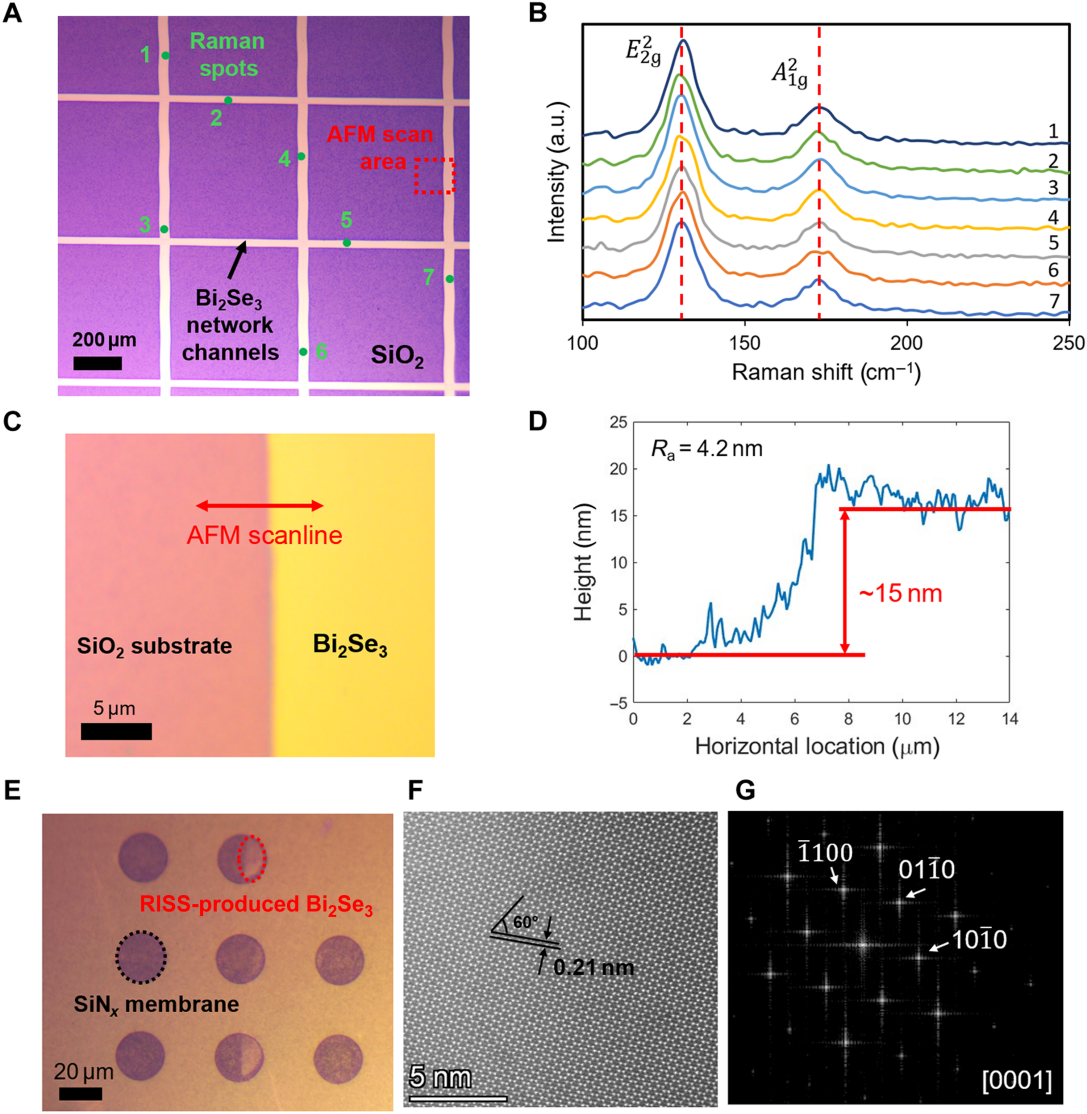
Material characterizations of the RISS-produced Bi_2_Se_3_ network for memristive RC applications. (**A**) OM image of a RISS-produced Bi_2_Se_3_ network in which the indexed points and the dashed box denote the locations for capturing Raman spectra and AFM images, respectively. (**B**) Raman spectra captured from the locations marked in (A), showing a small variance of FWHM and Raman shift values measured at their $E_{2g}^{2}$ peaks. a.u., arbitrary units. (**C**) Zoomed OM image of the AFM scan area marked in the dashed box in (A). (**D**) AFM scanline profile showing that the average thickness of the RISS-grown Bi_2_Se_3_ channels is ~15 nm and the surface roughness (*R*_a_) is ~4.2 nm. (**E**) OM image of another set of Bi_2_Se_3_ channels deposited on the SiN*_x_* membrane windows of an HRTEM sample grid under the same RISS processing condition. (**F**) HRTEM image of a representative RISS-produced Bi_2_Se_3_ channel marked in the dashed circle in (E), which shows that the interplanar spacing of the deposited crystal specimen is 0.21 nm and the angle between its principal planes is 60°. (**G**) FFT analysis of the HRTEM image, indicating the single hexagonal crystal structure of the deposited crystal specimen.

Figure 2A shows the optical micrograph (OM) of a representative Bi_2_Se_3_ network with an average channel width of 50 μm and an average channel spacing of 500 μm produced by the RISS process. The apparent color distribution of Bi_2_Se_3_ channels indicates a good continuity and uniformity of Bi_2_Se_3_ thickness over the whole network structure. These RISS-produced Bi_2_Se_3_ patterns also exhibit a high feature contrast between rubbed and unrubbed SiO_2_ surface regions. The uniformity of this Bi_2_Se_3_ network structure is further verified by Raman spectroscopy performed at seven different locations marked by the indexed points denoted in Fig. 2A. Figure 2B displays the Raman spectra captured at these locations. All of them feature with two characteristic peaks at Raman shifts of 130 and 173 cm^−1^, which are assigned to the in-plane vibration mode ($E_{2g}^{2}$) and out-of-plane vibration mode ($A_{1g}^{2}$) of the Bi_2_Se_3_ lattice, respectively. The full width at half maximum (FWHM) values and Raman shifts of the $E_{2g}^{2}$ peaks measured from these locations have relative variances of 2.1 and 3.2%, respectively, indicating a good uniformity of the Bi_2_Se_3_ thickness (*41*, *42*). Figure 2C displays the zoomed OM image of the sample area scanned by an atomic force microscope (AFM; Bruker ICON AFM, tapping mode), which is also accordingly marked by the dashed box in Fig. 2A. Figure 2D shows one of the exemplary AFM scanlines, which indicate that the average thickness of the RISS-produced Bi_2_Se_3_ network channels is ~15 nm and the surface roughness (*R*_a_) is ~4.2 nm. The same RISS process can also be performed to deposit Bi_2_Se_3_ channel features on 20-nm-thick silicon nitride (SiN*_x_*) membrane windows (purchased from Norcada Corporation) for high-resolution transmission electron microscopy (HRTEM) analysis. Figure 2E shows the OM of a RISS-deposited Bi_2_Se_3_ channel over several 20-nm-thick membrane windows, and the dashed circle indicates the location for capturing HRTEM images. The dark-field HRTEM image in Fig. 2F displays the RISS-produced Bi_2_Se_3_ lattice structure with an interplanar spacing of 0.21 nm and an angle between principal planes of 60°. Figure 2G displays the corresponding fast Fourier transform (FFT) analysis result, which further confirms its hexagonal structure with the zone axis [0001] and shows single-crystal characteristics of the specimen. Other details about the material characterizations are included in Materials and Methods.

### Electronic characterizations of the memristive network–based reservoir

**Fig. 3.**
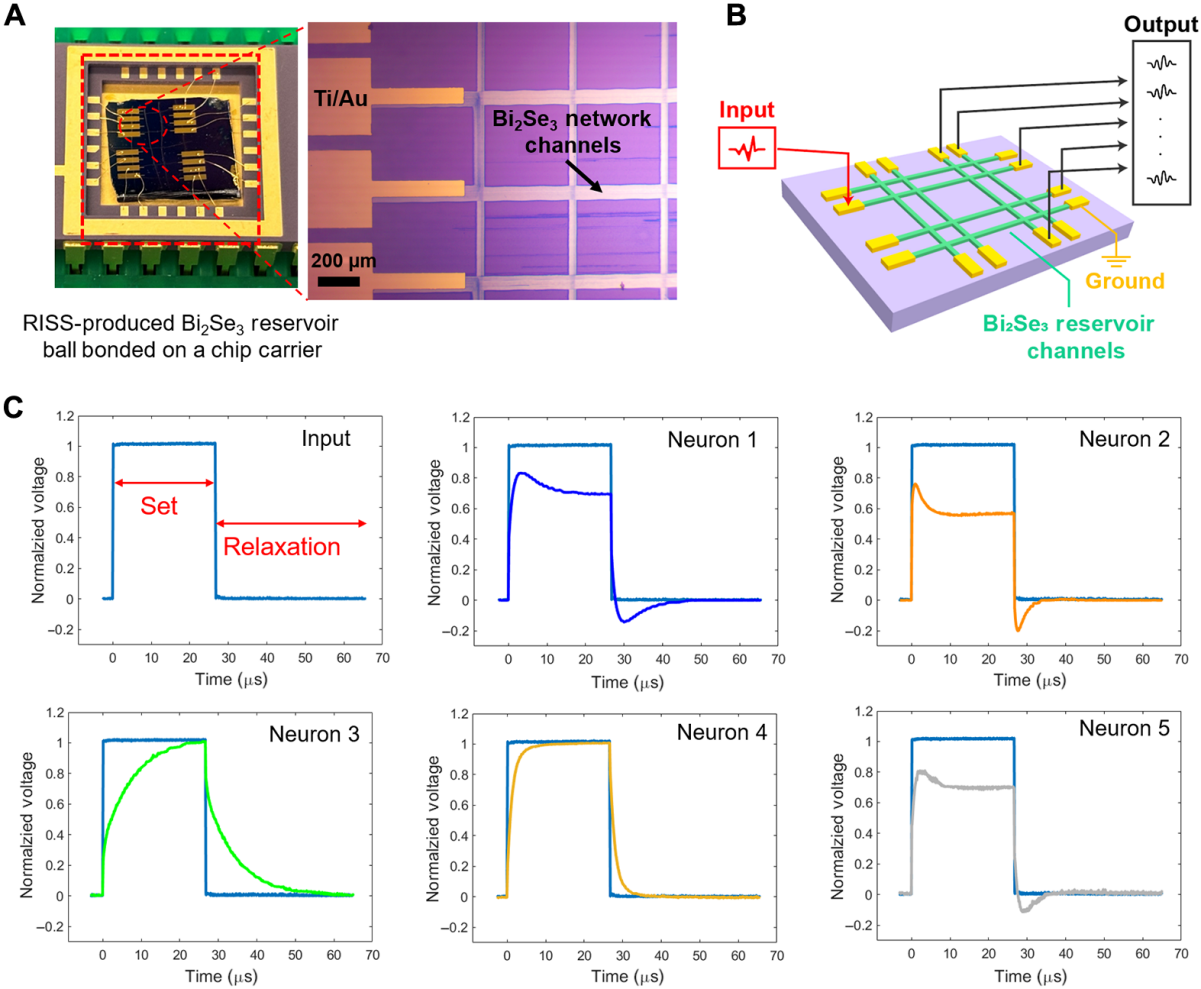
Response characteristics of the neurons in a Bi_2_Se_3_ memristive reservoir. (**A**) Photograph of a RISS-produced Bi_2_Se_3_ network wire bonded on a chip carrier through deposited electrode pads and the zoomed-in OM of the electrodes deposited on the dendrites of the network. (**B**) Schematic illustration of the Bi_2_Se_3_ reservoir layout including an input, a ground, and five output neuron terminals. (**C**) Normalized voltage response signals simultaneously measured at the five neuron terminals in response to the input square wave signal that is plotted with each response signal to show the diverse effects of the reservoir network on the resultant output signals through different neurons.

The RISS-produced Bi_2_Se_3_ network is ball bonded on a chip carrier through 16 deposited electrodes, as shown by the photograph in Fig. 3A. The zoomed OM view shows the electrodes connecting to the dendrites of the network, serving as input and output terminals of the memristive network–based reservoir. As schematically illustrated in Fig. 3B, for the electronic characterization, one of the dendrite terminals of the network serves as the input terminal and is connected to a sensory device, the other five terminals serve as the output terminals for dynamic reservoir state readout (i.e., five neurons are involved in this test), and another terminal is set as the common ground. To explore the dynamic response behaviors of the five neurons in this memristive network–based reservoir, we feed an 18-kHz square wave signal with an amplitude of 5 V and a duty cycle of 40% at the input terminal shown in Fig. 3B. Figure 3C displays the normalized voltage response signals simultaneously measured at the five neuron terminals in response to the input square wave signal. Here, all neurons exhibit nonlinear responses in their set stages [i.e., the 5-V (high) periods of the input square wave signal] and short-term memory behaviors in their relaxation periods [i.e., the 0-V (low) periods of the input square wave signal). More specifically, during the set [or 5 V (high)] periods of the input square wave signal, the voltage responses of neurons 3 and 4 increase exponentially with different time constants. This exponential rising behavior is a good fit for the sigmoid function that can be used as the activation function RC (*25*). Specifically, the logistic function, which is a typical sigmoid function, is expressed as the following equation

$$f\left(x\right)=\frac{a}{1+e^{-k\left(x-x_{c}\right)}}$$ (1)

where *a* is the carrying capacity for determining the amplitude of the function, *k* is the logistic growth rate for determining the steepness of the curve, and $x_{c}$ determines the value of the function’s midpoint. More details of the mathematic framework of the RC are included in the Supplementary Materials. Figure S1 shows that the voltage responses of neurons 3 and 4 can be well fitted with the logistic function. In addition, the response characteristics of neurons 1, 2, and 5 exhibit overshooting profiles, and this nonlinear inductor-like behavior can hardly be emulated by a traditional resistor-capacitor circuit. Various 2D layered materials, e.g., graphene, WSe_2_, and MoS_2_, have been reported to exhibit nonnegligible inductance effects, which involve magnetic inductance (*L*_M_) and kinetic inductance (*L*_K_) effects (*43*, *44*). Magnetic inductance is a property of an electrical conductor, and it quantifies the ability of the conductor in inducing electromotive force in itself or nearby conductors due to the instantaneous change of the current flowing through the conductor. Kinetic inductance arises from the inertia of charge carriers (typically electrons) in response to external electric fields. In a 2D material, the magnitude of kinetic inductance (*L*_K_) is comparable to that of magnetic inductance (*L*_M_) (*43*). Both of them could be responsible for the inductor-like behavior observed from the neurons in our Bi_2_Se_3_ reservoir network.

During the low periods (i.e., 0 V periods) of the input square wave signal, the voltage responses of the involved neurons relax back to their original states over different characteristic times, contributing to highly diverse short-term memory behaviors of the different neuron channels in the reservoir network. These diverse recovering times of the multiple neuron channels benefit the anticipated capability of the whole memristive network–based RC system for differentiating input sensory signals with different temporal contexts (i.e., current, historical, and trend readings). More specifically, these sensory signals can physically represent various kinetic states of a dynamic system. When input to the reservoir network, they can activate different dynamic state of the reservoir (i.e., collective resistive states of all interconnected memristive channels in the reservoir network). As implied by the results shown in Fig. 3, the voltage responses measured at multiple output terminals (neurons) of the network can form a time-sequential state vector signal for indicating the aforementioned dynamic state of the reservoir. Therefore, our Bi_2_Se_3_-based reservoir network is expected to enable direct mapping of the kinetic states of a dynamic system (represented by input sensory signals with different temporal contexts) to a high-dimensional data space (i.e., dynamic state space of the reservoir). This mapping process, once quantitatively determined, can be further correlated to the anticipated control signals through a simple machine learning course and ultimately realize energy- and resource-efficient controlling of a dynamic system.

Our work further shows that the aforementioned mapping functionality can be only obtained in the Bi_2_Se_3_ networks with a high degree of channel interconnection and complexity. For example, as shown in fig. S2A, a simple cross-network with one input and three neuron outputs is fabricated, and the output signals in response to the square wave signal are measured and plotted in fig. S2B. Because of the simplicity of this network, the voltage response signals measured from these three neuron outputs are highly similar to each other. This simple network structure does not have sufficient dynamic resistive states for differentiating the input signals with different temporal contexts. More discussion on the correlation between mapping functionality and required network structures is presented in the Supplementary Materials. In principle, any dynamic memristive devices with short-term plasticity behaviors could be used for constructing RC systems. However, our current research indicates that Bi_2_Se_3_-based memristive components exhibit unique nonlinear, short-term memristive switching characteristics, which are highly suitable for real-time analog signal processing. The typical memristive devices with long retention times or nonvolatile memory characteristics cannot spontaneously recover their conductance states for processing time-sequential signals, and, therefore, they are not suitable for RC applications.

### Memristive network–based RC system for robot rover control

**Fig. 4.**
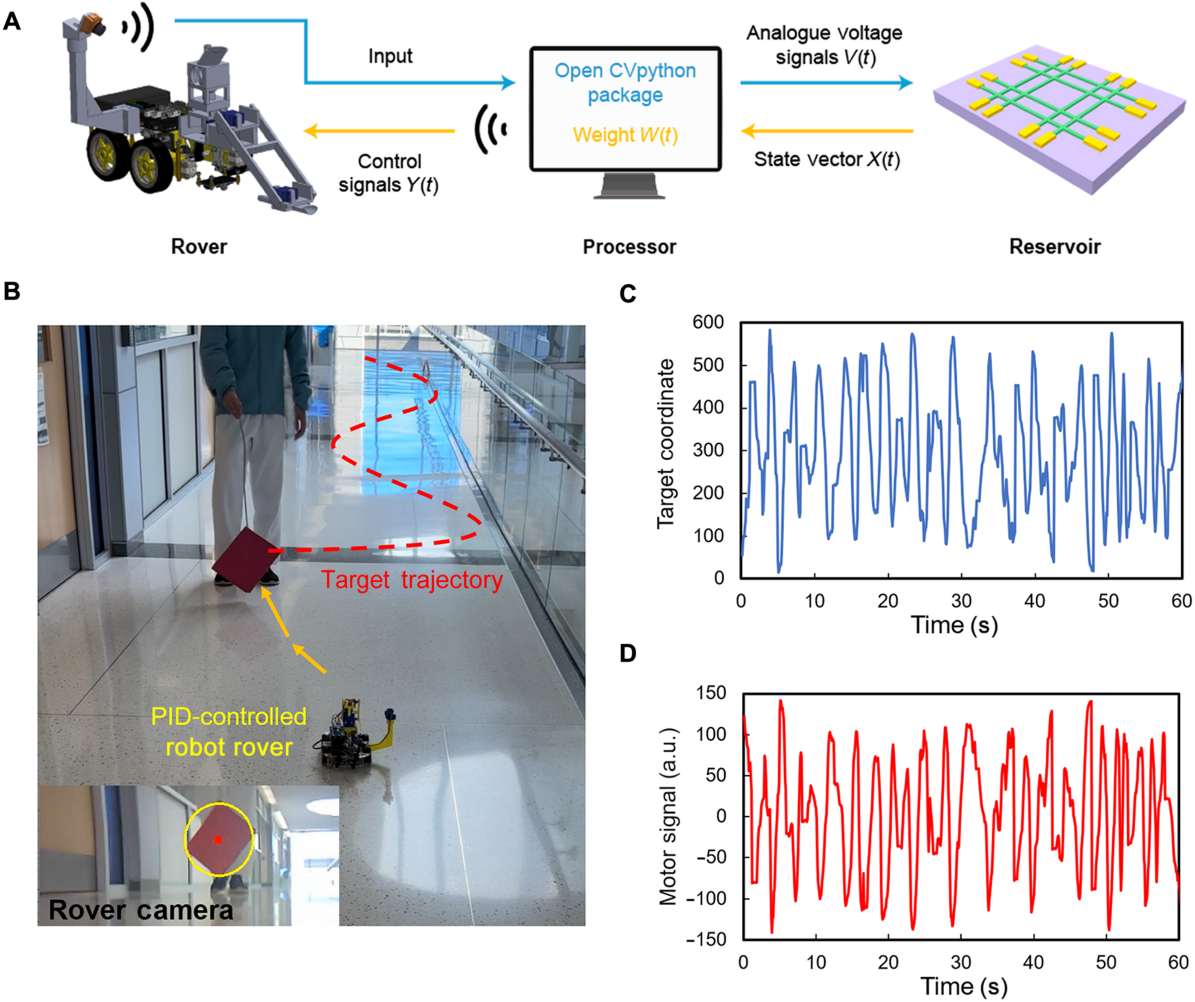
Target-tracking rover navigation task. (**A**) Schematic sketch for illustrating the implementation of the memristive network–based RC system for rover control through processing time-sequential sensory signals. Here, voltage-based analog sensory signals carrying spatiotemporal information components are input to the memristive reservoir. These input signals are differentiated and nonlinearly mapped to a high-dimensional data space based on the temporal contexts of the input sensory signals and are quantitatively represented by the reservoir state vector *X*(*t*), which is constructed from the voltage readings at multiple neuron terminals. Afterward, the state vector is multiplied by a pretrained weight matrix *W*(*t*) to export the output signals *Y*(*t*) for controlling the testing rover. (**B** to **D**) Training data acquisition for emulating PID control of a robot rover for performing target-tracking navigation: (B) snapshot captured from the training video, showing the PID-controlled rover tracing after a red-moving target (the inset view is a snapshot from the ESP32-based internet-of-things (IoT) camera on the rover); (C) exemplary target coordinate data plotted as the function of time points; (D) exemplary motor signal data generated by a digital PID controller, plotted as the function of time points.

Figure 4A illustrates the basic flow chart for implementing the memristive network–based RC system to control a robot rover for performing target-tracking navigation. First, the time-sequential sensor signal (i.e., the instantaneous coordinate of the tracked target captured by a computer vision system in this demonstration) is converted to an analog voltage signal *V*(*t*), which is applied to the input terminal of the reservoir. In the reservoir, the input signal (voltage) values with different temporal contexts can be differentiated and nonlinearly mapped to a high-dimensional data space, which is represented by the reservoir state vector *X*(*t*) constructed by the instantaneous voltage readings at multiple neuron output terminals. This state vector is subsequently multiplied by a pretrained weight matrix *W*(*t*) (i.e., a readout layer) to generate the expected output signal *Y*(*t*) for directly actuating the testing robot rover with a tank-like differential control scheme. Additional details about system integration and circuit configuration are included in Materials and Methods.

The aforementioned RC system can be trained to emulate the function of a conventional controller based on digital electronics, and the training process only seeks to create a proper readout layer [*W*(*t*)]. In this work, a digital PID controller, a feedback-driven control mechanism widely used in industrial control systems, is selected as the learning object for the memristive network–based RC system. In the training course, the PID controller is used to steer the testing robot rover to track a red target, which moves around in front of the rover (movie S1). Figure 4B shows a snapshot from the video of the training data acquisition course, and the dashed curve marks the motion trajectory of the red target in front of the testing rover. The inset image is the real-time video view from the onboard camera [i.e., an ESP32-based internet-of-things (IoT) camera], which, in combination with a computer vision program (i.e., opencv-python package), is capable of outputting the on-screen coordinate data of the red target. Figure 4C plots exemplary target coordinate data (ranging from 0 to 600) as a function of time points. Figure 4D plots the corresponding PID controller output signal values (ranging from −150 to 150) for steering the rover to track the red target, which is referred as the motor signal below. Specifically, in this data acquisition course, the rover’s motors are initially set to a constant autonomous navigation speed on a smooth floor surface. The motor signal value updated by the next target coordinate is added to the left-side motor speed rating (−255 to 255) and subtracted from the right-side motor speed rating (−255 to 255) for realizing differential control of the rover’s navigation direction. Here, the polarity sign of the instantaneous motor speed rating determines the rotation direction of the motor (or the wheel). Figure S3 shows a more detailed hardware setup diagram for this data acquisition process.

**Fig. 5.**
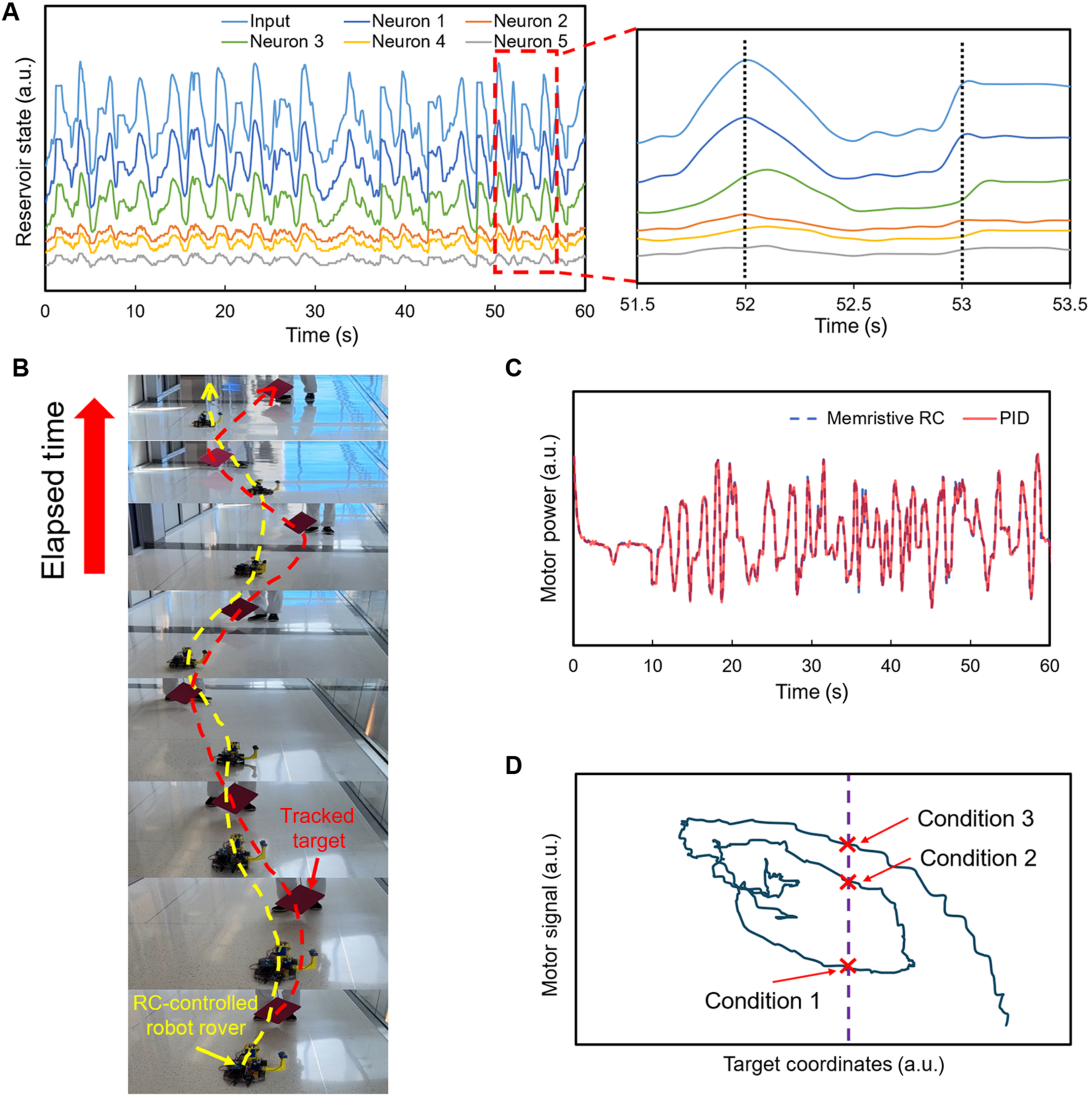
Training and testing the hardware-based RC system for rover navigation. (**A**) Electrical potential signals measured at five neuron output terminals in responses to the input signal derived from the target coordinate shown in Fig. 4C, representing the real-time state vector of the reservoir. The zoomed view of the regime denoted by the dashed box shows a prominent delay behavior for neurons 3 and 4 at time points of 52 and 53 s. This diverse delay behavior is consistent with the different short-term memory behaviors shown in Fig. 3C. (**B**) Snapshots captured from an RC testing video, arranged in time sequence, showing that the RC-controlled rover (with the trajectory marked by the yellow line) tracking after the motion of the red target (denoted by the red line). (**C**) Comparison between the motor signals generated by the original PID controller and the memristive RC system in response to an arbitrarily selected 60-s-long sensory signal, which exhibits a high degree of consistency. (**D**) RC system–generated motor signal values as the function of different coordinate values of the tracked target, measured in a navigation test. This result shows that the same location of the target can lead to different motor signal values, as demonstrated by conditions 1, 2, and 3. This implies that the output of an RC system depends not only on the current sensor signal value but also on the temporal contexts of the input signal.

The experimentally captured target coordinate data (Fig. 4C) and corresponding PID-generated motor control signal data (Fig. 4D) are subsequently used for training the readout layer for the RC unit. Specifically, the time-sequential coordinate data (ranging from 0 to 600) are linearly converted to an analog voltage signal ranging from 0 to 5 V using a general-purpose input/output port (fig. S4). This analog voltage signal is applied to the input terminal of the Bi_2_Se_3_-based memristive reservoir. At the same time, the resultant electric potential readings at the five neuron output terminals of the reservoir are recorded and plotted in Fig. 5A. These multichannel readings form the experimentally obtained reservoir state vector signal [*X*(*t*)]. The zoomed view of an exemplary regime marked by the dashed box in Fig. 5A shows that although all neuron output signals apparently show some extent of similarity in their peak-and-valley rhythms, they are not exactly synchronized with the input sensory signal. For example, around *t* = 52 and 53 s, neurons 3, 4, and 5 exhibit a prominent peak delay of ~0.1 s in comparison with neurons 1 and 2. These nonsynchronized temporal features occur frequently, implying the diverse and dynamic transport characteristics along different input-to-output pathways in the RC network. This network behavior, in combination with the short-term memory behaviors of involved Bi_2_Se_3_ memristive channels, facilitates the differentiation of the input sensory signals with varying temporal contexts.

The reservoir state vector data [*X*(*t*)] (Fig. 5A) electronically induced by the voltage-represented target coordinate signal data (fig. S4) are subsequently correlated with the PID-generated motor signal data (Fig. 4D) to train the software-based readout layer in our memristive network–based RC system. This training process was performed using an open-source Python library package (ReservoirPy). The trained readout layer is stored on a computer. A hardware-based readout layer could be also constructed using state-of-the-art NVM-based crossbar devices and integrated with our volatile memristive network to create an all-hardware RC system (*45*–*49*). Several recently published works have shown that NVM arrays are capable of processing analog signals through a linear multiplication process (*6*, *45*). The perpetual conductance values of these NVMs can serve as the matrix weights set in the readout layer. In the future, these NVM arrays could be integrated with our RC network to construct an all-hardware–based neuromorphic computing system.

The following procedure is for demonstrating the RC system–controlled rover navigation. First, as implemented in the training session, the ESP32-based IoT camera on the testing rover, in combination with the computer vision tool, keeps outputting real-time on-screen coordinate data of the tracked red target, which are converted to a voltage-based analog sensory signal carrying the temporal and kinetic information about the movement of the tracked target. This sensory signal is subsequently applied to the input terminal of the Bi_2_Se_3_-based reservoir for physically inducing the real-time reservoir state vector signal (i.e., diverse voltage signals from the five neuron output terminals). Because we use a software-based readout layer in this demonstration, the reservoir state vector signal is read and digitized through the analog pins of an Arduino board and input to the computer loaded with the trained readout layer. Last, the input reservoir state vector signal is multiplied with the weight matrix of the readout layer to generate the anticipated motor signal. Figure S5 shows the detailed flow chart of this signal processing procedure for RC-controlled target-tracking navigation. Movie S2 displays one of the demonstrations, and Fig. 5B shows the time-sequential snapshots captured from another demonstration video. As shown in movie S2 and Fig. 5B, the RC system–controlled rover exhibits a smooth and responsive moving behavior during the target-tracking navigation course, highly similar to the moving behavior mediated by the original PID controller. Figure 5C plots the motor signals generated by the original PID controller and the memristive RC system (with the trained readout layer) in response to an arbitrarily selected 60-s-long sensory signal, which exhibit a high degree of consistency and similarity. The NRMSE is used to quantitatively measure the difference between two sets of the signal data, which is described as

$$\text{NRMSE}=\sqrt{\frac{\sum {\left[Y_{\text{MRC}}\left(t\right)-Y_{\text{PID}}\left(t\right)\right]}^{2}}{\sum Y_{\text{PID}}\left(t\right)}}$$ (2)

where $Y_{\text{MRC}}\left(t\right)$ is the motor signal from the memristive RC system and $Y_{\text{PID}}\left(t\right)$ is the motor signal generated by the original PID controller. The NRMSE of our Bi_2_Se_3_-based RC system is calculated to be ~0.11 that is among one of the smallest NRMSE values ever reported for RC systems, strongly implying the good capability of our hardware-based RC system in emulating PID control functions and edge computing schemes for processing real-time sensory signals (*21*, *50*). In addition, Fig. 5D plots the RC system–generated motor signal data in response to different instantaneous coordinate data of the tracked target experimentally measured in a navigation test. Motor control signal values and target coordinates do not show a monotonical relationship. Specifically, as demonstrated by conditions 1, 2, and 3 denoted in Fig. 5D, the same target location (i.e., the same input sensory signal value) can lead to substantially different motor signal values. This observation indicates that a motor signal value generated by the memristive network–based RC system in combination with the readout layer depends not only on the current input sensory signal value but also on the temporal contexts of the input signal, which are manifested and differentiated by the dynamic characteristics of the memristive reservoir.

### Propeller-driven lever balancing for drone control

**Fig. 6.**
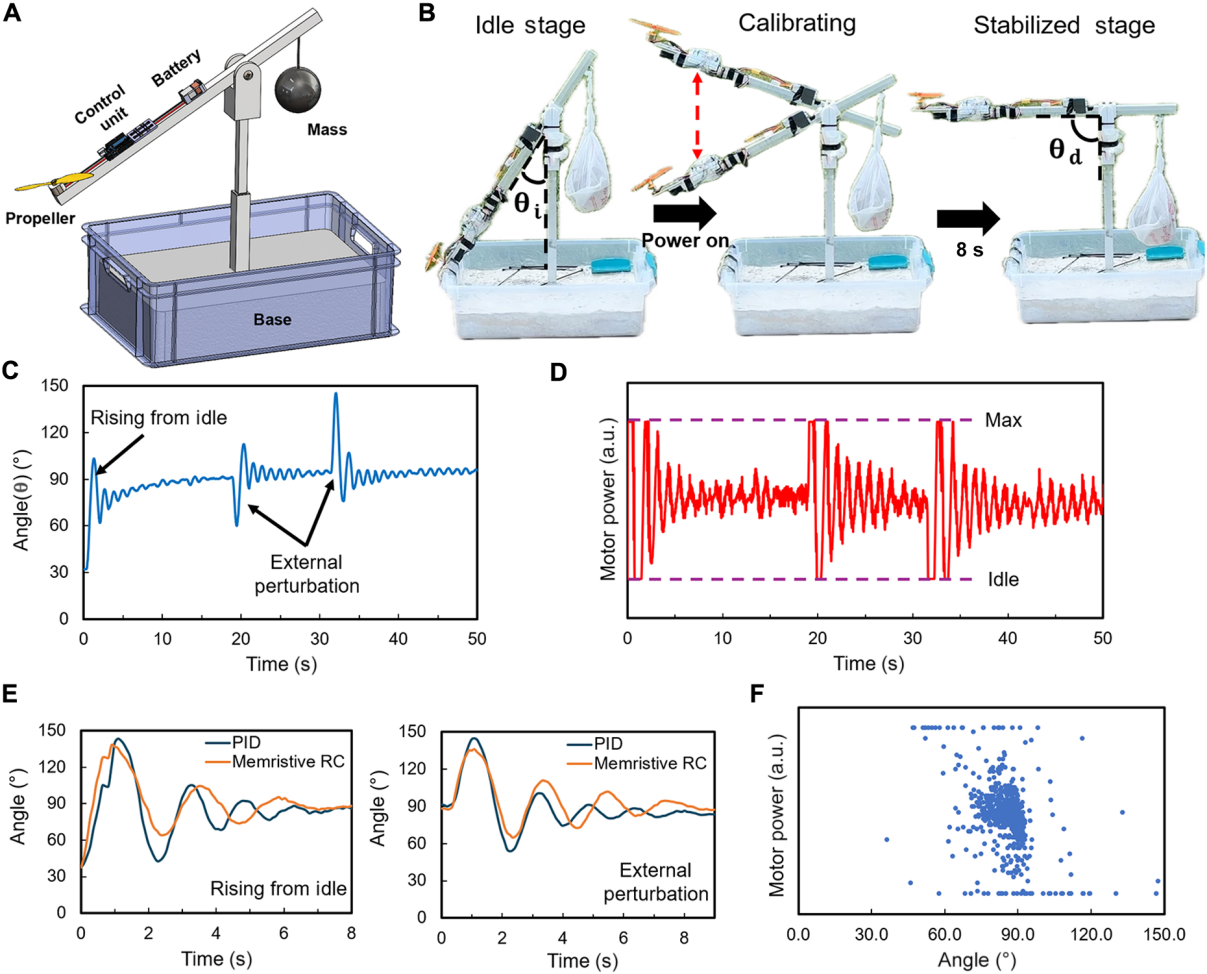
Training and testing the hardware-based RC system for lever balancing. (**A**) Schematic illustration of the testing rig, in which a drone motor with a propeller is mounted at the end of a lever for keeping the lever at the designated target position angle (θ). (**B**) Selected snapshot photos showing the motor-driven lever at three stages during a control test: At the initial idle stage, the lever naturally rests on the base. After the motor is turned on, the motor thrust, controlled by an RC- or PID-based controller, aims to set the lever position to the designed target angle of $\theta_{d}=90{}^{\circ}$. (**C**) Time-sequential lever position angle data showing a typical PID-mediated stabilization course of the motor-driven lever, in which several external perturbations are intentionally introduced to the system to acquire additional training data. (**D**) The PID-generated time-sequential motor power signal in response to the angle signal shown in (C). (**E**) The time-sequential lever angle signals measured from the control tests shown in movie S3, showing that our memristive RC system can balance the lever in a very similar way as the PID controller. (**F**) RC-generated motor power data plotted as the function of the instantaneous lever angle values, indicating that the output control signal values from the RC system depend on the temporal contexts of the input signals.

Our memristive network–based RC system is also implemented to control a drone motor with a propeller for keeping a lever in dynamic balance. The schematic illustration of this testing rig is shown in Fig. 6A, where the drone motor attached with a propeller is mounted at the end of a lever for supplying the lifting force, and the control unit of the motor is also mounted on the lever. A counter mass is placed at the other side of the lever to balance the weight of the control unit. The angle between the vertical supporting fixture and the lever is defined as the lever angle (θ). The devices in the control unit are wirely connected to each other, therefore minimizing the communication delay. Additional information about the circuit setup of the control unit can be found in Materials and Methods. Figure 6B displays three photos showing the lever located at three representative stages during a test. At the idle stage, the motor is off, and the lever end with the motor naturally rests on the rim of the base basket with an initial lever angle of $\theta_{i}=37.5{}^{\circ}$. To acquire training data, we power the motor through an electric speed controller (ESC) unit and dynamically control the thrust generated by the motor with an electronic PID controller, aiming to set the lever position to the designed target angle of $\theta_{d}=90{}^{\circ}$. During this PID-mediated control process, it takes approximately 8 s for the lever to be fully stabilized at the target angle. Here, the angular acceleration data of the lever, measured by a gyroscope mounted near the motor, are integrated by time steps to obtain the time-sequential lever angle data (or signal). A representative lever angle signal plotted in Fig. 6C shows a typical PID-mediated stabilization course of the motor-driven lever. After the initial lever stabilization, several external perturbations are intentionally introduced to the system to acquire additional training data for describing the recovering behaviors of the PID controller in response to various external disturbances. At the same time, the corresponding PID-generated motor power signal is recorded and plotted in Fig. 6D. After the collection of the PID data, the lever angle signal (Fig. 6C), which is converted to a voltage signal (fig. S6A), is applied to the memristive network, and the resultant reservoir state vector signal is recorded by measuring the induced voltage signals at all neuron output terminals, as plotted in fig. S6B. Similar to the readout layer used for the rover navigation test, a weight matrix is trained to correlate the reservoir state vector data (fig. S6B) with the expected motor power data (Fig. 6D). Movie S3 is a real-time video, which compares the control performance of RC- and PID-based controllers in initial stabilization of the motor-driven lever and dynamic responses to external perturbations.

Figure 6E plots the time-sequential lever angle signals measured from these two testing courses shown in movie S3. These results show that our memristive RC system can balance the lever in a very similar way as the PID controller. For both tests, it takes ~8 s to stabilize the lever at the designed position angle, and the NRMSE value between these two control processes is calculated to be 1.25. This NRMSE value is reasonably good for indicating the dynamic similarity between these two control systems. It is higher than that measured from the rover tests probably because the rover navigation test is performed indoor with a well-controlled environment condition, while the lever balancing test is performed outdoor with unpredicted environment conditions (e.g., wind speed and temperature changes). The test of a trained RC system can hardly be performed under the same environmental condition as that for acquiring the training data. Therefore, the prediction difference between the PID controller and the RC system is inevitable. More training data acquired under different environmental conditions are anticipated to reduce this discrepancy. In addition, the lever system has a much smaller damping coefficient in comparison with the rover system, which makes the lever system more sensitive to the external perturbations in comparison with the rover system. In addition, Fig. 6F plots the RC-generated motor power data as the function of the instantaneous lever angle values obtained from a lever balancing test. This plot also shows that the same lever angular position can be mapped to very different motor power values. This further indicates that the output control signals from our memristive RC system largely depend on the temporal contexts of the input signals. These results could be further leveraged for making ultrasmall RC–based flight controllers for mini-sized drones. More details about the experimental setups are presented in Materials and Methods. Our experiments indicate that the RC device can still properly function after hundreds of rover navigation or balancing lever tests in several weeks. Even if the conductance values of the network channels change over time, we can easily recover the RC system by retraining the weight matrix of the readout layer with the established dataset, which only takes a few seconds.

## DISCUSSION

### Power consumption analysis

One of the highly expected advantages of hardware-based RC networks is the low power consumption needed for data processing. To estimate an upper bound for the power consumption of our Bi_2_Se_3_-based reservoir, we assume that the input sensory signal values remain at the maximum value (i.e., $V_{\text{max}}=5\;V$), and the average output current for each neuron channel is experimentally measured to be $I_{\text{max}}=5\;\mu A$ for both rover navigation and lever balancing tests. Therefore, the upper bound of the power consumption of the reservoir is estimated to be ~12.5 μW using the following equation

$$P_{\text{PR}}=I_{\text{max}}\times V_{\text{max}}\times N_{\text{PR}}\times t_{p}\times f_{s}$$ (3)

where $N_{\text{PR}}$ is the number of the output neurons used in the task ($N_{\text{PR}}=5$), $t_{p}$ is the readout pulse duration ($t_{p}=1\;\text{ms}$), and $f_{s}$ is the signal sampling rate ($f_{s}=10\;\text{Hz}$). As a comparison, the software-based PID control is typically performed on a single-board reduced instruction set computer microcontroller (e.g., Arduino Nano), with an operating voltage of 5 V and an average output current of ~1 mA. Therefore, the power consumption of a digital PID system is ~5 mW, which is about 400 times higher than that of our memristive network–based RC system. In addition, to our knowledge, the power consumption of our device is among one of the lowest power values reported for hardware-based RC systems, as shown in Table 1.

**Table 1.** Comparison with literature-report memristor-based RC system.

Works	Reservoir type	Readout device	Tasks	Power consumption
Moon *et al.* (*33*)	DM	Computer	Speech recognition	300 μW
Yu *et al.* (*58*)	Ferroelectric tunneling junctions	Resistive random-access memory (RRAM)	Digital sequence classification	70 μW
Milano *et al.* (*8*)	Nanowire networks	RRAM	Pattern recognition	75 mW
Zhong *et al.* (*21*)	DM	Computer	Spoken digit recognition	50 μW
Zhong *et al.* (*6*)	DM	RRAM	Dynamic gesture recognition	22.2 μW
**This work**	**Memristive network**	**Computer**	**Rover navigation**	**12.5 μW**

We also compare several important parameters of our memristive network–based RC system to those of recently reported RC systems based on NVMs, as shown in Table 2. This table displays that the RC systems based on nonvolatile memristive or memory devices have power consumptions in the range of 15 to 287 μW for various tasks (*51*–*55*). In contrast, our Bi_2_Se_3_-based dynamic memristive network system demonstrates a lower power consumption of 12.5 μW in real-time control tasks such as rover navigation and lever balancing. Nevertheless, the networks based on NVMs are effective for static tasks involving long-term data storage. The DMs in our RC networks provide short-term plastic memory characteristics, which are highly suitable for processing temporal signals and controlling dynamic systems, offering a substantial advantage for real-time robotic applications.

**Table 2.** Comparison with literature-report NVM-based RC system.

Works	Reservoir type	Readout device	Tasks	Power consumption
Lin *et al.* (*51*)	NVM	Computer	In-memory and in-sensor RC	15 μW
Ryabova *et al.* (*54*)	Parylene-MoO × crossbar memristor	RRAM	Homogeneous RC system	50 μW
Jang *et al.* (*53*)	Nonvolatile optoelectronic memristors	Computer	High-dimensional machine vision	100 μW
Chen *et al.* (*52*)	Graphene oxide films	Computer	Flexible RC	120 μW
Tsioustas *et al.* (*55*)	SiO_2_-based memristor	Computer	Handwritten digit recognition	287 μW

### Response time and noise analysis

The response time of the RC system is quantitatively evaluated by measuring the average time delay between an input voltage–based sensory signal and the resultant voltage readings at all five neuron channel terminals. Figure S9 shows the zoomed voltage response at a representative neuron terminal (i.e., neuron 3), where the output signal exhibits a time delay of 0.06 μs in comparison with the input rising signal. The average response time measured from all five neuron terminals is 0.055 μs, which can be regarded as the response time of our RC network in processing temporal signals. The response time of the digital PID controller is evaluated by measuring the elapsed time of each iteration loop using the built-in function “*millis()*”. For the lever balancing test, the average loop time is ~6 ms, which is approximately 10^5^ times slower than the average response time of our network-based RC system.

To evaluate the read noise from the memristive RC network, we first evaluate the signal-to-noise ratio (SNR) values at the input and output terminals of the network. Figure S11 shows the raw voltage data of a square wave signal applied at the input terminal and the response signal measured at a representative output terminal. The average SNR of the input signal is measured to be 41.7 dB, and the average SNR of the output response signals is measured to be 31.9 dB. Therefore, the noise generated in the memristive network remains at a relatively low level (*56*, *57*).

In summary, we present a Bi_2_Se_3_-based memristive network device for performing RC and demonstrating the rover navigation and motor-driven lever balancing processes controlled by this device, which exhibit low power consumption. In this work, the relationship between the input sensory signals and induced reservoir state changes of the Bi_2_Se_3_ network has been studied and quantified using the voltage responses of all neuron channels. The diverse short-term memory behaviors of these neuron channels facilitate extraction of the temporal information components from the input signals. Using these device characteristics, we demonstrated RC-based control of a robot rover for performing target-tracking navigation and a motor-driven lever for dynamic balancing. In comparison with the traditional PID control scheme based on digital electronics for performing the similar tasks, this hardware-based RC system enables a much lower power consumption ($P_{\text{PR}}=12.5\;\mu W$) and a small prediction error (e.g., NRMSE = 0.11 for the rover navigation test and NRMSE = 1.25 for the lever balancing test), indicating that for these dynamic control tasks, RC-based controllers exhibit very similar response behaviors as PID controllers. This work provides a prototype device, which could be further leveraged for constructing powerful hardware platforms of RC and other RNN schemes. This energy- and resource-efficient hardware platform also paves a pathway for miniaturization of robotic vehicles. Moreover, the training data generated from different control algorithms, including force control, adaptive control, and model predictive control, could be used to train the readout layers for enabling a broader range of RC applications.

## MATERIALS AND METHODS

### Fabrication of the Bi_2_Se_3_-based memristive reservoir network

The Bi_2_Se_3_ network is fabricated using our previously reported RISS deposition method (*34*). Briefly, an Au-coated Si template bearing a 50-μm-wide protrusive pillar is brought into contact with a Si/SiO_2_ substrate loaded on a 3D moving stage system. The contact stress between the protrusive pillar and the substrate is precisely controlled by the *z*-direction stage. The substrate carried by the *x*-*y* stage moves in a predefined path to generate a triboelectric charge network pattern when the pillar on the template rubs the SiO_2_ surface of the substrate. The rubbed substrate with the triboelectric charge network pattern is loaded into a tube furnace set at 550°C for 7 min to selectively deposit Bi_2_Se_3_ network features within the rubbed SiO_2_ surface areas (i.e., the areas with triboelectric charge) (*34*, *35*). Subsequently, a set of 500-nm-thick Ti/Au finger electrodes is deposited at the dendrites of the network. The as-fabricated Bi_2_Se_3_ reservoir network is subsequently ball bonded in a dual in-line sidebraze package with a cavity of 0.38 inch^2^ (2.45 cm^2^) (Souza Semiconductor Materials) using an MPP iBond 5000 Wedge Bonder. The dual in-line package is placed on a breadboard and connected to other electronic units through jumper wires.

### Material characterization

Raman scattering spectroscopy is performed on a Renishaw inVia Raman microscope with a spectral resolution of 0.5 cm^−1^. The 532-nm laser is applied on the sample with an illumination power of ~5 mW. The AFM characterization is performed on a Bruker ICON AFM tool using the tapping mode with a ScanAsyst-Air probe. The scan rate is 0.7 Hz, and the total scanned area is 20 μm by 20 μm. The thickness of the Bi_2_Se_3_ network structures mainly depends on the duration time of the vapor deposition process. Our experimental results indicate that a 7-min deposition process results in a Bi_2_Se_3_ thickness of 15 nm, which is the optimal Bi_2_Se_3_ thickness in terms of resultant diversified memristive responses through different neuron channels.

The HRTEM characterization is carried out on a Thermo Fisher Scientific Spectra 300 Probe-Corrected S/TEM tool with an acceleration voltage of 300 kV. To prepare the Bi_2_Se_3_ samples for HRTEM characterization, a rubbing template bearing 10-μm-wide square pillars is used to perform the RISS process on a silicon nitride TEM grid with a membrane thickness of 10 nm (Norcada Inc.). The same deposition process as the one for producing Bi_2_Se_3_ memristive network devices is performed on the rubbed TEM grid to obtain RISS-produced Bi_2_Se_3_ channel features for HRTEM analysis.

### Training for the readout layer of the RC system

To acquire the training data from a regular PID controller for performing target-tracking navigation of a robot rover, a red cardboard is used as the tracking target (more detailed explanation and schematic diagram of this PID controller are presented in the Supplementary Materials and fig. S10). During a data acquisition course, a researcher holds the red target and randomly swings it in front of a PID-controlled robot rover. In this rover, a PID firmware program flashed on an Arduino Nano microcontroller is used for controlling the rover direction to follow the tracking target. An ESP32-CAM camera module mounted on this PID-controlled rover wirelessly broadcasts the real-time video streaming signal to a laptop where a Python-based computer vision program is activated to locate the red target location on the received video frames and constantly sends the on-screen coordinate values of the tracking target back to the PID-controlled rover also through the ESP32-CAM module. Once the updated coordinate data of the tracking target are received, the PID firmware codes on the Arduino controller of the rover generate an updated motor signal for actuating the rover motors. During this course, time-sequential data of target coordinates and motor signal values are recorded and synchronized for the readout layer training. Afterward, a function generator (SIGLENT Technologies, SDG1032X) is used to convert the recorded time-sequential target coordinate data to an analog voltage signal, which is applied to the input channel of the Bi_2_Se_3_ memristive network–based reservoir. At the same time, the five output neuron terminals of the Bi_2_Se_3_ memristive network–based reservoir are connected to a multichannel oscilloscope (SIGLENT Technologies, SDS1104X-E) for recording the real-time electric potentials at these neuron channels, which represent the reservoir states of the Bi_2_Se_3_ network. Last, an open-source Python library (ReservoirPy) is used to train the weight matrix for correlating the reservoir state vector data to the anticipated motor signal values.

On the basis of the mathematical principle of RC, different tasks require different numbers of neuron channels. For a given task, insufficient neurons cannot offer enough dynamic network states for mapping and differentiating temporal information components carried by the input signals, while excessive neurons could lead to overfitting of training data. In this work, for each control task, we first operate software-based RC networks with different numbers of neurons using a Python-based software package (ReservoirPy). On the basis of the performance of each reservoir network in terms of prediction accuracy and reproducibility, we can determine the optimal number of neurons for the specific task. Here, a neuron channel is defined as a pathway that comes through the input and one of the output terminals. Although the neurons in a network have different memristive response behaviors, they may share some pathways.

### Target-tracking navigation controlled by the RC network

During a rover navigation test, the ESP32-CAM camera module on the testing robot rover keeps wirelessly sending video stream to a controlling laptop. The Python-based computer vision program on this laptop keeps processing the received video stream signal and extracting the horizontal coordinate of the tracking target in the view (ranging from 0 to 600), which is being constantly converted to an analog voltage signal in the range of 0 to 5 V using a 12-bit digital-to-analogue converter (DAC) (MCP4725). This analog signal, carrying the location information of the target, is sent to the input channel of the Bi_2_Se_3_-based reservoir network for performing hardware-based RC and generating the reservoir state vector signal, which is formed by instantaneous electrical potential values induced at multiple neuron terminals of the network. A set of analog pins of an Arduino microcontroller is connected to these neuron terminals and keeps sampling the electrical potential values at these terminals. This measured reservoir state vector signal is sent to the controlling laptop where the pretrained weight matrix of the readout layer is used to generate the output motor signal. The generated motor signal is wirelessly sent back to the rover’s motor driver for steering the navigation direction.

### Motor-driven lever balancing

Figure S7 shows a photo of the testing rig for motor-driven lever balancing. The whole lever system is anchored on a 50-pound (22.68-kg) concrete base. A brushless drone motor with a propeller is mounted at one end of the lever and connected to the three power output terminals of an ESC board. The power input port of this ESC unit is connected to a 12.6-V lithium polymer battery, and the control signal input port of the ESC is connected to a pulse-width modulation (PWM) signal output pin of an Arduino microcontroller. A three-axis accelerometer gyroscope module (MPU6050) and a 12-bit DAC unit (MCP4725) are mounted on the lever and also electrically connected to the serial clock line and serial data line pins of the Arduino microcontroller. The output of the DAC unit is connected to the input terminal of the memristive RC network. The output and ground neuron terminals of our memristive RC network are connected to the analog pins (input) and the ground pin of the Arduino microcontroller, respectively. These control units are placed close to the pivot of the lever to minimize their inertial effects. In addition, a 500-g counterweight is placed at the other end of the lever to balance the moment generated by the aforementioned components. Figure S8 displays the flow chart for implementing our memristive network–based RC system to control the motor thrust for balancing the lever. During a balancing course, the gyroscope module sends out the angular acceleration signal to the Arduino microcontroller, where the acceleration signal is integrated over time to generate the angle signal. This lever angle signal is converted to an analog voltage signal through a 12-bit DAC and directly applied to the input terminal of our Bi_2_Se_3_-based memristive network. A set of analog pins of an Arduino microcontroller reads out the voltage signals at those neuron terminals, which compose the reservoir state vectors of the network. This state vector signal is synchronously processed by the pretrained weight matrix prestored in the Arduino microcontroller to generate the PWM signal for controlling the motor power, seeking to stabilize the lever at the target angle.
