# Supplementary material for: Nanoelectronics-enabled reservoir computing hardware for real-time robotic controls
Source: Sci Adv. 2025 Mar 26;11(13):eadu2663. doi: 10.1126/sciadv.adu2663 (PMC11939057; doi:10.1126/sciadv.adu2663)
Supplement: Supplementary file 1 — Supplementary Text S1 to S3 Figs. S1 to S11 Legends for movies S1 to S3 [file sciadv.adu2663_sm.pdf]

Science Advances
AAAS

Supplementary Materials for

Nanoelectronics-enabled reservoir computing hardware for real-time robotic controls

Mingze Chen *et al.*

Corresponding author: Xiaogan Liang, xiaoganl@umich.edu

Sci. Adv. **11**, eadu2663 (2025)
DOI: 10.1126/sciadv.adu2663

The PDF file includes:

Supplementary Text S1 to S3
Figs. S1 to S11
Legends for movies S1 to S3

Other Supplementary Material for this manuscript includes the following:

Movies S1 to S3

Supplementary text 1: Mathematical Framework of Reservoir Computing

A generic mathematical formula for reservoir computing is expressed as follow (*24*):

$$y(n) = f_{out}[W_{out} \cdot x(u(n))] \quad \text{(S1)}$$

Where y(n) is the output result; f_{out} is a nonlinear activation function (*e.g.,* an elementwise sigmoid function); W_{out} is the weight matrix. Function *x(u(n))* nonlinearly transforms an input *u(n)* into a higher-dimensional vector x(n), which represents the reservoir state vector induced by the input *u(n)* and is corresponding to the voltage readings physically measured at the output neuron terminals of our hardware-based RC system. For temporal tasks, the *x(u(n))* is replaced by the time-dependent function: *x(u(n-t), ..., u(n))*, which incorporates the historical or temporal context information carried by the input signals.

In our RC system, the weight matrix (W_{out}) is implemented by a Python-based software (reservoirpy). The reservoir computing network state (*x(n)*) is physically represented by the voltage readings measured at all neuron terminals (*i.e.*, a network state vector). Here, *u(n)* is the input time-sequential analog voltage signal applied at the input neuron terminal. The activation function f_{out} and the nonlinear transformation function *x(n)* are physically implemented through the nonlinear memristive response behaviors of the Bi_2Se_3 networks.

Supplementary text 2: Algorithm of a PID Controller

Proportional-Integral-Derivative (PID) control algorithms is a widely used feedback-based (or closed-loop) control scheme that constantly adjusts the output control signal of a dynamic system to minimize the error between the desired setpoint of the system state and the actual system state with the optimal tradeoff between system overshooting and relaxation time. A PID controller is set to realize this controlling objective through adjusting and balancing three key components: the proportional unit (P), the integral unit (I), and the derivative unit (D), as illustrated in Fig. S10.

Proportional Unit (P): This unit addresses the current error by generating an output control signal proportional to the error. It ensures an immediate correction because the larger error results in the larger correction. The proportional gain K_p regulates how sensitively the dynamic system reacts to the instantaneous error.

Integral Unit (I): This unit accounts for the historic accumulation of previous errors over time sequences. Its role is to eliminate steady-state errors which the proportional unit alone cannot resolve. The integral gain K_i regulates the rate at which the steady-state error is corrected, but an excessively large K_i may lead to the overshooting or instability of the system.

Derivative Unit (D): This unit predicts the future error by monitoring the rate of change of the error (i.e., trend of error). It facilitates the reduction of overshoot and damping oscillations,

leading to a smooth response or relaxation behavior for the dynamic system. The derivative gain K_d determines the characteristic relaxation time of the dynamic system. An excessively large K_d may result in slow relaxation behaviors for the system in response to external perturbations.

For our lever balancing task, a high proportional unit (K_p) can decrease the rising time of the lever, but it can also enhance the overshoot which features large oscillations of the lever. The integral unit (K_i) is adjusted to eliminate the steady-state error of the lever angle, which may come from the mechanical design or environmental conditions. A properly set derivative unit (K_d) can lead to a reasonable damping coefficient of the system and also increase the stability of the lever under external perturbations. By appropriately tuning K_p, K_i, and K_d, a PID controller can be tailored to achieve an optimal tradeoff among stability, response speed, and steady-state error for the dynamic system under study.

The mathematic formula for describing the PID control algorithm and the relevant schematic diagram (Fig S10.) are shown below:

$$u(t) = K_p\theta(t) + K_i\int_0^t \theta(t)dt + K_d\frac{d\theta(t)}{dt} \qquad (S2)$$

Where *u(t)* is the control signal (*e.g.,* motor speed), $\theta(t)$ is the difference between the current lever angle and the set angle (error at time), K_p is the proportional gain, K_i is the integral gain and K_d is the derivative gain.

Supplementary text 3: Diversified Mapping Capability and Network Structures

The diversified mapping capability, an important device aspect for enabling a successful RC network, is hypothesized to be highly related to the structure complexity of the network. In this work, such a complexity is mainly determined by the number of interconnections in the network pattern. To support this hypothesis, we have fabricated a simple Bi_2Se_3 network with only a single interconnection, as schematically illustrated in **Fig S2 (a)**. When a square-wave voltage signal is applied at the input terminal of this network, the voltage readings measured at its three output neuron terminals (**Fig. S2 (b)**) exhibit a high degree of similarity, which results in a poor mapping capability for the network to map and differentiate the temporal components carried by the input signal. In addition, we hypothesize that other 2D Bi_2Se_3 networks with a high degree of structure complexity (or an adequate number of interconnections) can also function as effective RC systems. Supplementary **Fig S2 (c)** presents two network patterns with more than 15 interconnection nodes which have potential to enable effective diversified mapping capability comparable to that of the presented RC network with a grid-like structure.

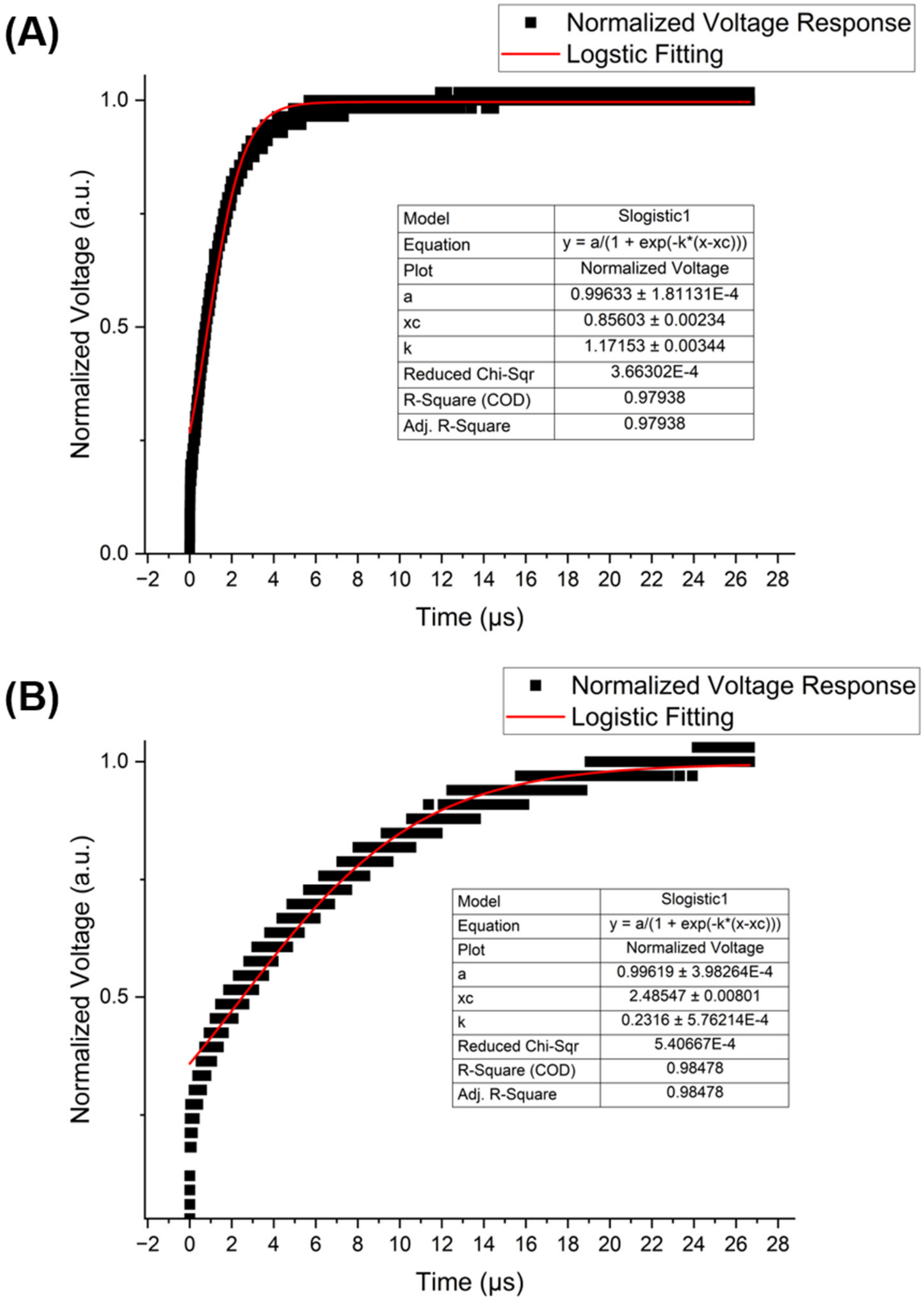

Fig. S1. **Logistic fitting for the normalized voltage responses measured from two representative neuron terminals.** (A) Response signal measured from Neuron 3; (B) signal measured from Neuron 4. The inset table shows the corresponding fitting parameters and error.

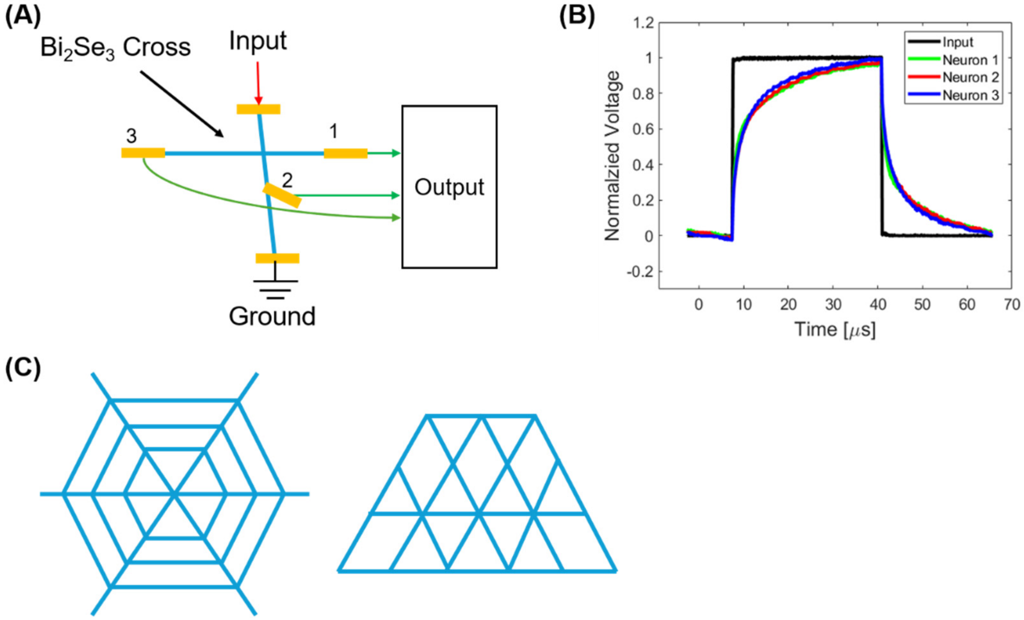

Fig S2. Response characteristics of a single network and other proposed reservoir computing network structures. (A) Schematic illustration of a simple Bi_2Se_3 network with only a single interconnection and 3 output neuron terminals; (B) The normalized voltage readings measured at the three output neuron terminals of this network in response to the input square-wave voltage signal, which shows highly similar dynamic response characteristics among these three neurons; (C) Two proposed network patterns with potential to enable diversified mapping capability similar to that of the presented grid-like network.

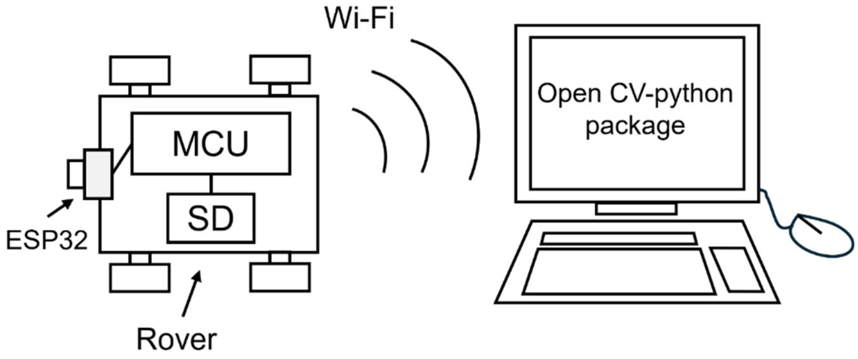

Fig. S3. Schematic illustration of the training data collection system for the target-tracking rover navigation test. It consists a robotic rover carrying an ESP32-based video server and a control computer installed with the OpenCV-Python software package.

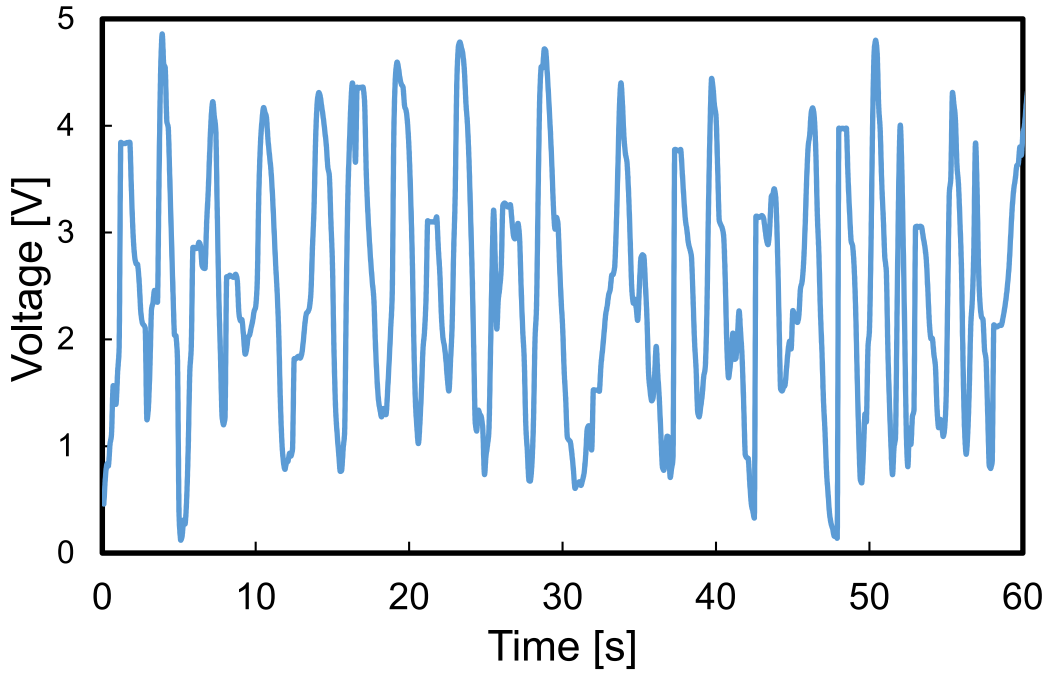

Fig. S4. Time-sequential voltage signal converted from the target coordinate data shown in Fig. 4(c). This is the analogue signal physically applied to the Bi_2Se_3 reservoir computing network.

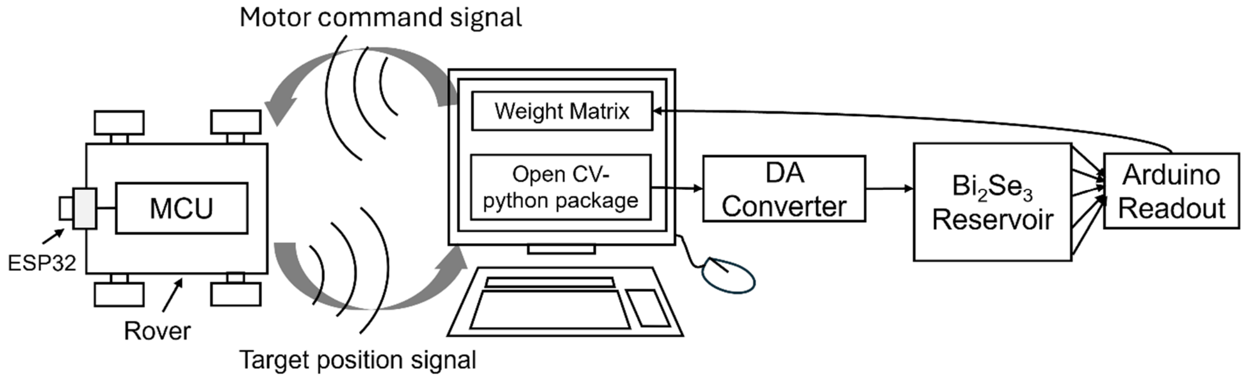

Fig. S5. Schematic illustration of the signal processing procedure in the RC-guided navigation test. It consists a robotic rover carrying an ESP32-based video server, a control computer installed with the OpenCV-Python software package and the trained weight matrix of the readout layer, a digital-to-analog (DA) converter, and the Bi_2Se_3 reservoir computing network chip interfacing with an Arduino controller.

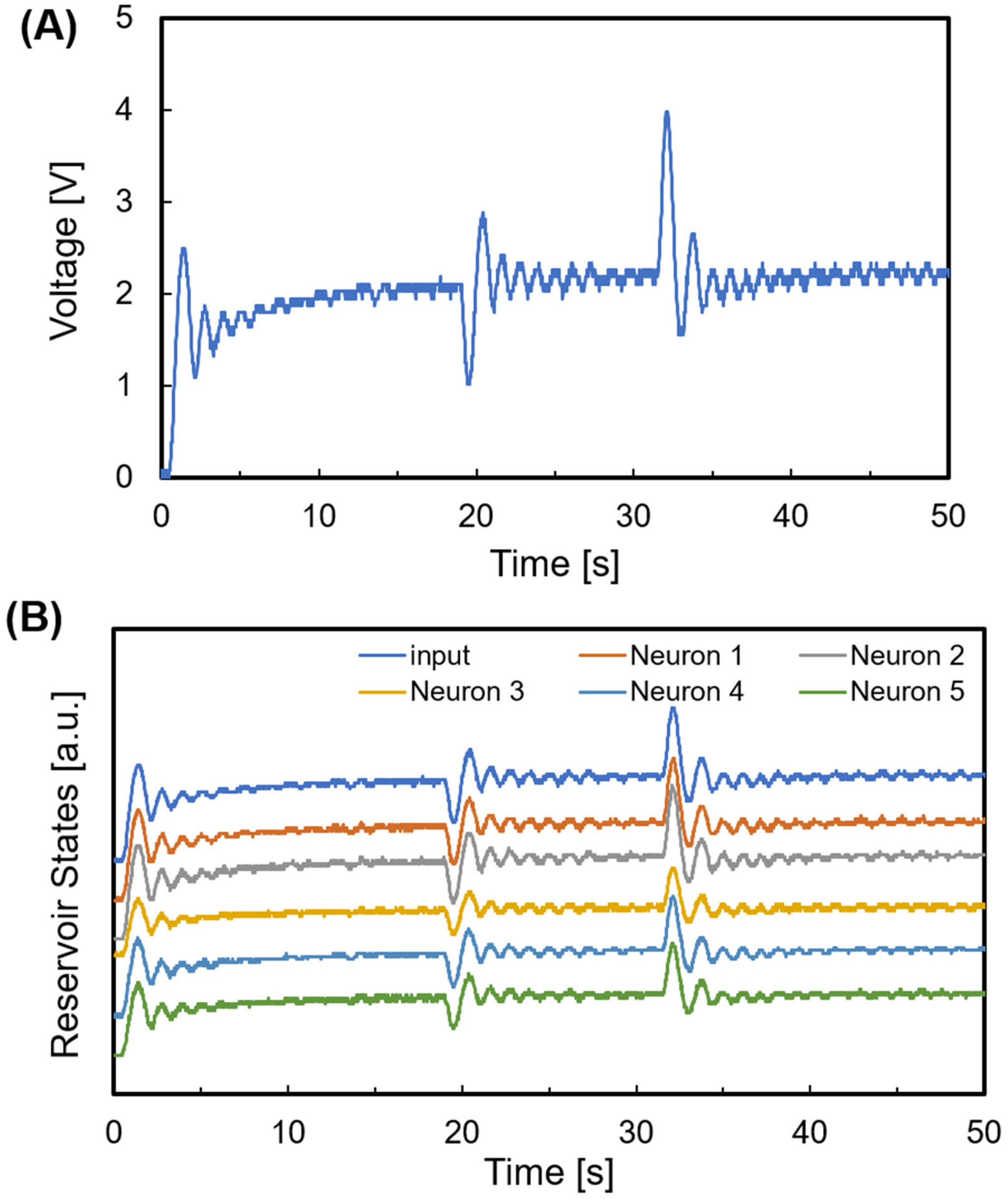

Fig. S6. The reservoir state vector signal acquired in the lever balancing test. (A) Time-sequential voltage signal converted from the lever angle signal shown in Fig. 6C; (B) The reservoir states of the memristive reservoir compared with the input signal.

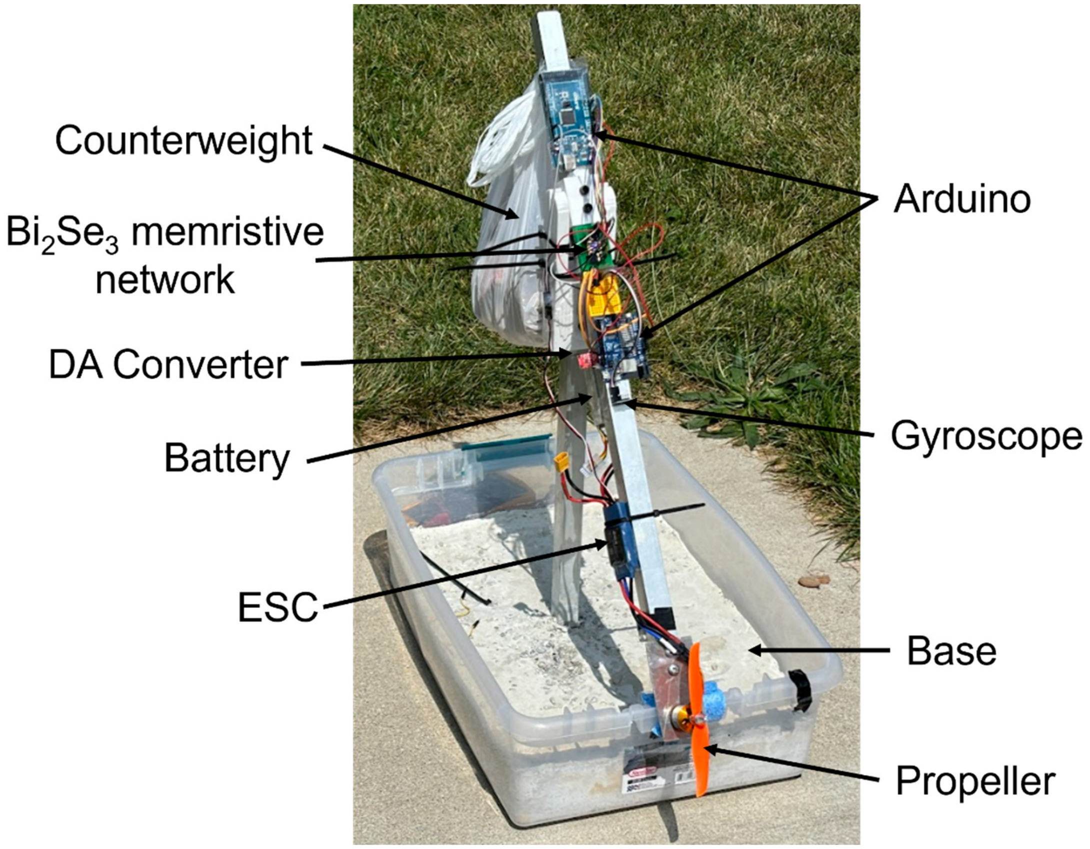

Fig. S7. Photograph of the testing rig for the motor-driven lever balancing test. The whole system is placed in an outdoor environment for carrying out the test.

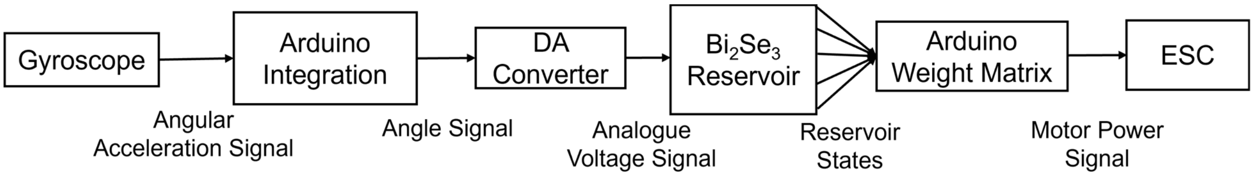

Fig. S8. Flow chart for implementing the memristive network-based RC system to control the motor thrust for balancing the lever. The whole system consists of a gyroscope for measuring the real-time angular acceleration of the swing lever, an Arduino controller for capturing the lever angle signal, a DA converter for generating the analogue voltage signal for activating the Bi_2Se_3 reservoir, another Arduino module that stores the trained weight matrix of the readout layer, and an electric speed controller (ESC) that is regulated by the control signal from the reservoir/readout layer module.

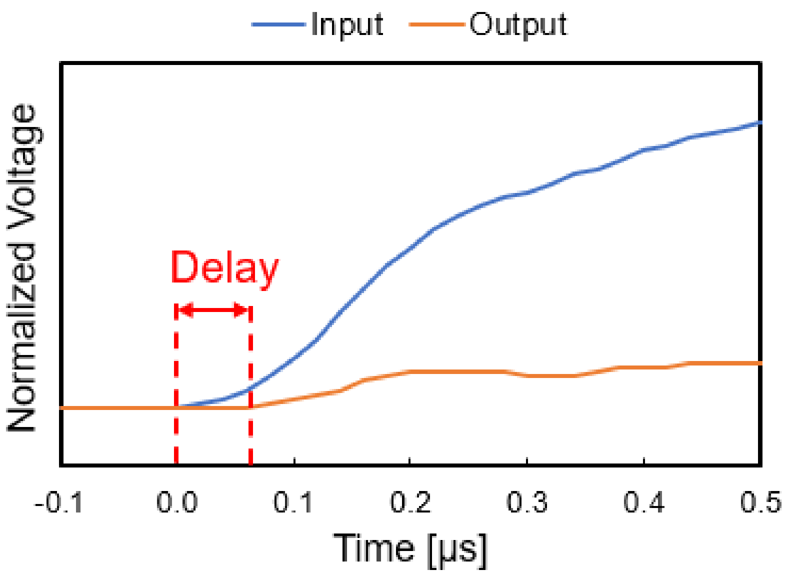

Fig. S9. Zoomed voltage response of a representative neuron terminal (i.e., Neuron 3). This result shows a response delay of ~ 0.06 μs.

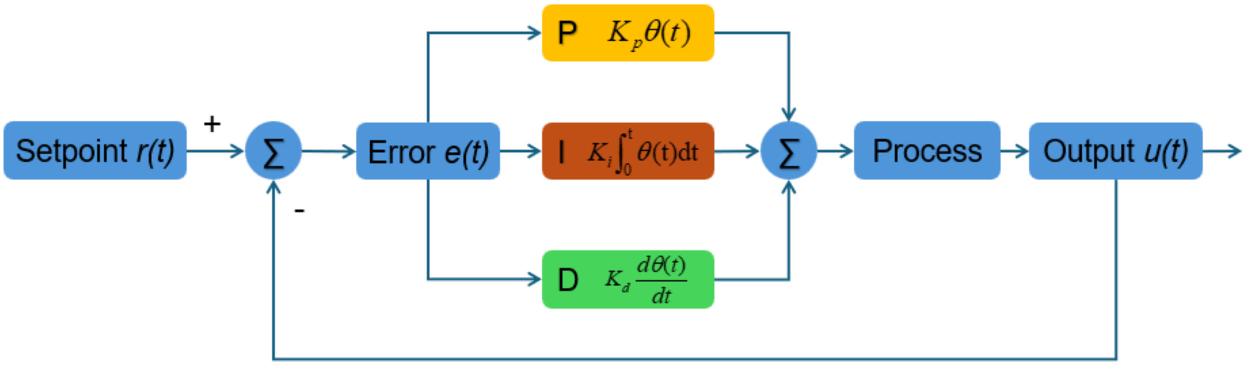

Fig. S10. Block diagram of a PID control loop. In this work, this control scheme is physically emulated by the presented Bi_2Se_3 reservoir network system.

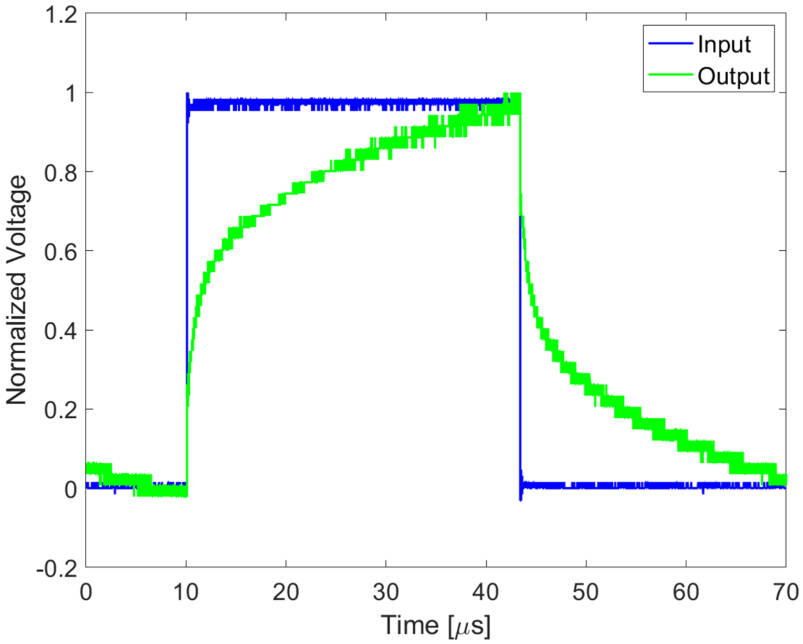

Fig. S11. Analysis of the noise added by the reservoir computing network. This graph shows the raw voltage data of a square-wave signal applied at the input terminal of the RC network and the response signal measured at a representative output terminal. The memristive network introduces additional noise to the output signals, but the average SNR of the output signals is still in a reasonable range.

Supplementary Movies

Movie S1. Demonstration of the training data acquisition for rover navigation task.

Movie S2. Demonstration of the memristive network-controlled rover accomplishing the navigation task.

Movie S3. Real-time video comparing the control performance of the RC-based and PID-based controllers for the lever balancing task.
